# Using community pharmacies to expand access to screening for noncommunicable diseases in suburban Ghana—A facility‐based survey on client needs and acceptability

**DOI:** 10.1002/hsr2.79

**Published:** 2018-07-30

**Authors:** Richard Akutey, Reina Der, Frances Owusu‐Daaku, Frank Baiden

**Affiliations:** ^1^ Epidemiology Unit Ensign College of Public Health Kpong, E/R Ghana; ^2^ Department of Pharmacy Practice Kwame Nkrumah University of Science and Technology Kumasi Ashanti Region Ghana; ^3^ Faculty of Infectious and Tropical Diseases London School of Hygiene & Tropical Medicine London UK

**Keywords:** Africa, community, Ghana, noncommunicable diseases, pharmacy, screening

## Abstract

**Background:**

Many of the 28 million deaths from noncommunicable diseases (NCDs) in low‐ and middle‐income countries each year could be prevented through early detection and intervention. The introduction of screening for NCDs in community pharmacies (CPs) in Ghana could enhance access to early detection.

**Methods:**

We surveyed clients in three districts in suburban Ghana to assess perceived need for screening, willingness to be screened in CPs, and willingness to receive NCD health promotion information through text messages (NCD m‐Health). We performed regression analysis to identify predictors of NCD m‐Health acceptability.

**Results:**

We interviewed 330 clients in six CPs, 134 (42.3%) of whom were females. The median age was 34 years (interquartile range, 27‐43). Fifty‐four (16.4%) had no formal education. Although most respondents knew obesity (74.9%), smoking (81.9%), and excessive dietary salt (91.7%) were risk factors for NCDs, only 27.0% knew family history carried similar risk. Most respondents, 61.6% and 70.6%, respectively, had not had their weight and blood pressure (BP) checked for more than 12 months. These included about a third of respondents who were known hypertensives. Similarly, 71.3% of 80 participants with a family history of hypertension had not had their BPs checked. Screening for NCDs in CPs and the sending of NCD m‐Health messages was deemed acceptable to 98.5% and 83.1% of the participants, respectively. Formal education beyond junior high school (Grade 9) was the strongest independent predictor of NCD m‐Health acceptance (OR = 4.77; 95% CI, 1.72‐13.18; *P* value < 0.01). One hundred and twenty‐five (39.4%) participants indicated they would consider unsolicited NCD m‐Health messages an invasion of their privacy.

**Conclusion:**

An urgent need exists to promote access to NCD screening in these communities. Its introduction into CPs is acceptable to nearly all the clients surveyed. The introduction of NCD m‐Health as an accompaniment requires consideration for the privacy of clients.

## 1 BACKGROUND

Noncommunicable diseases (NCDs) kill 38 million people each year, with almost three quarters of these deaths occurring in low‐ and middle‐income countries (LMICs). 1 The catastrophic cost associated with long‐term treatment entrenches poverty and undermines productivity. It is predicted that by 2020, NCDs will cause seven out of every 10 deaths in these countries.^2^ Without purposeful implementation of a global plan to tackle NCDs in LMICs, Sustainable Development Goal (SDG) 3, which targets reduction in premature NCD mortality by a third by 2030, is not likely to be achieved.^3^, ^4^, ^5^, ^6^

In 2010, hypertension occurred to 31.1% of the world's adult population, 28.5% (27.3%‐29.7%) in high‐income countries and 31.5% (30.2%‐32.9%) in LMICs.^7^ Studies in Ghana and Nigeria in West Africa and Lesotho and rural Zulu in South Africa have found hypertension prevalence rates of between 15% and 50%.^8^, ^9^, ^10^ There is the need to identify simple, low‐cost interventions that will address the high burden of hypertension in these countries in sub‐Saharan Africa (SSA).^1^ As a major NCD, it is estimated that tens of millions of hypertension‐related deaths could be prevented through BP checking and early care.^11^

The four main types of NCDs are cardiovascular diseases (such as heart attacks and stroke), cancer, chronic respiratory diseases (such as chronic obstructed pulmonary disease and asthma), and diabetes. These NCDs share smoking, excessive alcohol, lack of physical activity, and poor eating habits as common modifiable risk factors.^12^, ^13^ The majority of NCDs develop slowly. This makes screening (where possible) an important intervention in their control, especially in LMICs where access to curative services is limited.

The primary health care (PHC) systems of most countries in SSA evolved in response to the impact of communicable diseases. The exact form of a health system in SSA that adequately responds to the epidemic of NCDs remains a subject of continuing research. An important agenda in this regard is identifying the appropriate platform for expanding access to screening and primary care for high‐risk individuals, while appropriately managing treatment options in resource‐limited settings.^14^, ^15^, ^16^

One of the most decentralized structures within the health system of many countries in SSA is the operation of community pharmacies (CPs). Community pharmacies are found to operate in settings where other health services such as clinics and hospitals are not available.^17^ For a variety of reasons, community members often prefer to make CPs their first port of call for disease prevention advice and medicines to initiate treatment.^18^ The potential for the use of CPs to expand opportunities for NCD screening is gradually being appreciated.^19^, ^20^ In most of SSA, however, this is yet to be formalized and systematically deployed as an intervention in NCD prevention. A systematic review of the evidence on the feasibility and acceptability of using CPs to screen for major diseases concluded that screening for some diseases in CPs was generally feasible.^21^ Questions remain about acceptability in different sociocultural settings, cost effectiveness, and the nature and form of follow‐up services.

In Ghana, NCDs are now recognized as major causes of significant illness and deaths. The evidence base for community‐based interventions is weak because NCDs have, until recently, not been a part of PHC in the country.^22^, ^23^, ^24^ The health system in Ghana is in acute need of evidence‐based interventions that will guide expanded access to NCD screening, early detection, and management.^25^, ^26^ The accessibility of CPs and their widespread use as a port of first contact makes them potentially ideal for NCD screening and related primary care services. The regulated nature of community pharmacy practice in Ghana implies that the integration of NCD screening in CPs can be formalized and supervised. Evidence on the potential role of m‐Health in providing low‐cost follow‐up services is also emerging.^27^

As part of an intended program to introduce screening for NCDs in CPs in three districts in southeastern Ghana, we conducted a survey among clients of CPs in these districts to assess acceptability and to identify other possible threats to effective implementation.

## 2 METHODS

### 2.1 Study design and site

The study was conducted in CPs in the Asuogyaman, Lower Manya Krobo, and Yilo Krobo districts of the Eastern region of Ghana. The three districts have a combined population of about 400 000. Most inhabitants live in rural settlements. The main occupations are subsistence farming and fishing. The most common ethnic groups are the Ga‐Adangbes and Akwamus. The respective district capitals, Akosombo, Odumase, and Somanya, are all suburban. Each district has at least either a district hospital (Asuogyaman, Lower Manya Krobo) or a polyclinic (Yilo Krobo) that are staffed by a medical officer and where comprehensive services, including special clinics and surgeries, are performed.

The public health services in the three districts are managed by the District (and Municipal) Health Management Teams. The approach to the delivery of public health services is based on the PHC concept, with emphasis on the control of communicable diseases and provision of maternal and child health services. The delivery of adult health services including NCD screening remain largely at the level of health centers and hospitals at subdistrict and district levels, respectively. The most decentralized form of health service provision are privately owned CPs and over‐the‐counter–medicine shops. There is a total of nine CPs in the three districts.

Over a 6‐week period, adult (aged ≥18 y) clients at six selected CPs in the three districts were interviewed using a questionnaire that inquired into sociodemographic background, personal and family history of hypertension and diabetes, access to screening for hypertension and diabetes, and willingness to avail oneself of CP‐based screening services and to receive NCD‐related health promotion messages via text messaging (m‐Health). The input of the owners of CPs, experienced local public health practitioners, and program officers of community‐based health programs were sought to finalize the questionnaire.

The most highly patronized CPs in the three districts were selected, and the owners were approached for permission to interview clients. At the time of the study, none of the selected CPs was offering NCD screening services. All adult clients reporting to the CPs during the day were targeted to be interviewed. The interviews were conducted by trained research assistants and in dialects that clients were comfortable to speak in. Most of the questions on the questionnaire were close ended.

The data were entered into a computer using a platform created in Microsoft Access 2013. Double data entry was used to ensure accuracy. Data were then exported into Stata (version 12, College Station, Texas) for analysis. Sociodemographic variables were analyzed descriptively using chi‐square and means. Proportions and percentages were computed based on the number of respondents who agreed to respond that specific question. Willingness to be screened and to receive m‐Health messaging via text were analyzed using logistic regression. We included in the multivariate logistic regression model variables that were significant in bivariate analysis at a *P* value of less than 0.05. The final model was obtained using backward elimination procedures. The results of regression analysis are presented here as odds ratios (OR) with 95% confidence intervals (CI). All *P* values are derived from chi‐square analysis except in instances when contingency tables contain numbers that are less than five. In such instances, Fisher's exact estimates are reported.

### 2.2 Sample size

We planned to enroll 401 respondents on the basis that it will afford an estimation of the proportion of clients willing to be screened for NCD within a margin of error of 3.8% at 95% confidence level, assuming 80% of clients will consider it acceptable. The predicted level of acceptability was based on the finding of an acceptance level of 70% in a study in Ghana where actual testing was performed.^27^ The six selected CPs see about 12 000 clients in a year. The target sample size was not achieved due to logistical constraints (see below).

### 2.3 Ethical approval

The protocol for the study was reviewed and approved by the Institutional Review Board of the Ensign College of Public Health, Kpong, Ghana. Individual informed consent was obtained from each participant prior to the start of interview. The request to participate in the survey was made only after clients had been served at the CPs and were about to exit. No information that identified individual clients was obtained.

## 3 RESULTS

Overall, 352 clients were approached in six CPs over a 6‐week period between March and May 2016. Out of this number, 330 (93.5%) agreed to be interviewed. The main reason given by clients who refused to participate was time constraint. Interviewed clients consisted of 134 (42.3%) females. The median age was 34 (interquartile range, 27‐43) years. The majority were Christians, 292 (89.9%). Just about half, 148 (44.89%) were married. Most of the clients (169, 51.2%) had formal education beyond junior high school, while 54 (16.4%) had no formal education. About half of the clients (160, 48.5%) belonged to the indigenous ethnic group Ga‐Adangbe. Most respondents (181, 55.5%) lived more than an hour's walking distance to the pharmacy at which they were interviewed; only 43 (13.2%) lived within 30‐minute walking distance. About a quarter, 62 (22.6%) of clients were unemployed at the time of the interview (Table 1).

**Table 1:** Bivariate analysis to determine how respondents' sociodemographic background predict agreement to receive NCD m‐Health promotional mobile phone messages

Variable		n	Agree to Receive NCD m‐Health Messages		Unadjusted		Adjusted Odds Ratio (95% CI)
			Yes	No	Odds Ratio (95% CI)	*P* value	
Sex	Male	183 (57.7)	141 (88.9)	40 (22.1)	0.44 (0.23‐0.86)	0.01	0.39 (0.18‐0.83)
	Female	134 (42.3)	112 (88.9)	14 (11.1)	1.00		1.00
Age, y	>50	39 (11.8)	33 (84.6)	6 (15.4)	0.36 (0.12‐1.13)	<0.01	0.39 (0.09‐1.62)
	31‐50	158 (47.8)	111 (73.5)	40 (26.5)	0.18 (0.08‐0.42)		0.22 (0.07‐0.61)
	≤30	133 (40.3)	122 (93.9)	8 (6.2)	1.00		1.00
Religion^a^	Christian	292 (89.9)	232 (82.0)	51 (18.0)	0.14 (0.02‐1.12)	0.04	NA
	Non‐Christian	33 (10.2)	31 (96.9)	1 (3.1)	1.00		
Ethnicity^a^, ^b^	Ga‐Adangbe	160 (49.5)	129 (82.7)	27 (17.3)	0.57 (0.16‐2.04)	0.79	NA
	Ewe	65 (20.1)	50 (80.7)	12 (19.4)	0.50 (0.13‐1.97)		
	Akan	68 (21.1)	55 (82.1)	12 (17.9)	0.55 (0.14‐2.15)		
	Other	30 (9.3)	25 (89.3)	3 (10.7)	1.00		
Marital status	Married	148 (44.9)	116 (80.1)	28 (19.4)	0.45 (0.22‐0.92)	0.03	1.47 (0.56‐3.88)
	Divorced/separated/widowed	48 (14.6)	34 (72.3)	13 (27.7)	0.29 (0.12‐0.69)	<0.01	0.65 (0.20‐2.13)
	Single	13 (40.6)	119 (90.2)	13 (9.9)	1.00		1.00
Highest educational level	SHS/graduate	169 (51.2)	147 (90.7)	15 (9.3)	3.19 (1.38‐7.37)	<0.01	4.77 (1.72‐13.18)
	Primary/JHS	107 (32.4)	79 (75.2)	26 (24.8)	0.99 (0.46‐2.13)		1.70 (0.69‐4.15)
	None	54 (16.4)	40 (75.5)	13 (24.5)	1.00		1.00
Walking distance: Residence to pharmacy, min	>60	181 (55.5)	153 (86.9)	23 (13.1)	1.29 (0.51‐3.26)	0.08	NA
	30‐60	102 (31.3)	74 (76.3)	23 (23.7)	0.63 (0.24‐1.60)		
	<30	43 (13.2)	36 (83.7)	7 (16.3)	1.00		
Employment status	Unemployed	62 (22.6)	51 (83.6)	10 (16.4)	0.91 (0.42‐1.99)	0.82	NA
	Employed	212 (77.4)	173 (84.8)	31 (15.2)	1.00		

Abbreviations: CI, confidence interval; JHS, junior high school; NA, not applicable; NCD, noncommunicable disease.

a Excluded from the multivariate model because of small numbers

b Fisher exact estimates of *P* values. Multivariate model was significant at the *P* value of <0.01.

### 3.1 Awareness of risk factors and willingness to be screened for NCDs

Obesity, smoking, and excess dietary salt were known to 245 (74.9%), 266 (81.9%), and 299 (91.7%) of clients, respectively, as risk factors for developing hypertension and diabetes. The fact that family history was a risk factor for these diseases was much less known (88, ie, 27.0%). Only 112 (35.1%) clients knew their weight at the time of the interview. The last time 197 (61.6%) of the clients had a weight check was over 12 months ago. Similarly, only 67 (20.8%) had an idea about what their usual blood pressure (BP) level was. For 203 (70.6%) respondents, the last time they had their BP checked was over 12 months ago (Table 2).

**Table 2:** Bivariate analysis to determine how knowledge, health‐seeking behavior, and family history predict agreement to receive NCD m‐Health promotional mobile phone messages

Variable			n	Agree to receive NCD m‐Health Messages		Unadjusted		Adjusted Odds Ratio (95% CI)
				Yes	No	Odds Ratio (95% CI)	*P* value	
Knowledge of the risk factors for NCDs	Obesity	Yes	245 (74.9)	192 (80.3)	47 (19.7)	0.40 (0.17‐0.93)	0.03	0.32 (0.13‐0.79)
		No	81 (24.7)	72 (91.1)	7 (8.9)	1.00		1.00
	Family history	Yes	88 (27.0)	67 (79.8)	17 (20.2)	0.74 (0.39‐1.41)	0.36	NA
		No	238 (73.0)	196 (84.1)	37 (15.9)	1.00		
	Smoking	Yes	266 (81.9)	211 (82.1)	46 (17.9)	0.72 (0.32‐1.62)	0.43	NA
		No	59 (18.2)	51 (86.4)	8 (13.6)	1.00		
	Excess dietary salt^a^	Yes	299 (91.7)	240 (83.0)	50 (17.2%)	0.83 (0.28‐2.52)	1.00	NA
		No	27 (8.3)	23 (85.2)	4 (14.8)	1.00		
Knowing one's weight	Yes		112 (35.1)	87 (84.5)	20 (18.7)	0.70 (0.37‐1.32)	0.27	NA
	No		207 (64.9)	174 (86.1)	28 (13.9)	1.00		
Most recent weight check	>1 y ago		197 (61.6)	154 (80.6)	37 (19.4)	0.65 (0.34‐1.24)	0.25	NA
	≤1 y ago		123 (38.4)	102 (86.4)	16 (13.6)	1.00		
Knowing one's BP	Yes		67 (20.8)	57 (90.5)	6 (9.5)	2.15 (0.87‐5.33)	0.09	NA
	No		255 (79.2)	203 (81.5)	46 (18.5)	1.00		
Most recent BP check	>12 mo ago		230 (70.6)	179 (80.0)	45 (20.1)	0.43 (0.20‐0.93)	0.03	0.45 (0.20‐1.03)
	<12 mo		96 (29.4)	83 (90.2)	9 (9.8)	1.00		1.00
Family history of hypertension	Yes		82 (25.0)	63 (77.8)	18 (22.2)	0.51 (0.23‐1.14)	0.25	NA
	No		142 (43.3)	114 (83.2)	23 (16.8)	0.73 (0.35‐1.53)		
	Don't know		104 (31.7)	88 (87.1)	13 (12.9)	1.00		
Family history of diabetes	Yes		44 (13.4)	31 (70.5)	13 (29.6)	0.33 (0.14‐0.79)	0.03	0.69 (0.26‐1.81)
	No		158 (48.2)	126 (82.9)	26 (17.1)	0.67 (0.34‐1.34)		0.99 (0.44‐2.21)
	Don't know		126 (38.4)	108 (87.8)	15 (12.2)	1.00		1.00

Abbreviations: CI, confidence interval; NA, not applicable; NCD, noncommunicable disease.

a Fisher exact estimates of *P* values. Multivariate model was significant at the *P* value of <0.01.

Nearly all, 322 (98.5%) and 312 (95.7%) clients, agreed with the proposal to have BP measurement and urine testing for sugar introduced into CPs. Over half of the respondents, 184 (55.8%) and 187 (56.7%), thought that the provision of BP and urine screening services at CPs should be free of charge. Of those who thought the services should be performed for a fee, the mean costs suggested were GHC 1.29 and GHC 4.48 (equivalent of USD 0.33 and USD 1.13) for BP and urine screening, respectively. Forty‐four (13.5%) also indicated they had close blood relations who had diabetes. Only 46 (13.9%) respondents indicated they were current or former tobacco smokers.

### 3.2 Weight and BP check for high‐risk clients

Thirty‐four (10.5%) clients indicated they were hypertensive. Out of this number, however, about a third, 11 (32.3%) and 12 (35.3%), had not had either their weight or BP checked for more than 12 months. Eighty‐two clients (25.2%) also indicated they had blood relations who were hypertensive. Out of this number, 51 (62.2%) involved either or both parents. For more than 12 months prior to the survey, 47 (57.5%) and 58 (71.3%) of clients with a family history of hypertension had not had either their weight or BP checked. More than half, 67 (67.0%) and 41 (60.4%), of clients aged 40 years or more could neither tell what their usual BP levels were nor the last time they had it checked. Clients who were older than 50 years of age were more likely to know the usual BP levels than those ages less than 30 years (chi‐square test *P* value = 0.04). There was, however, no such statistically significant association between older age (>50 y) and BP checking (chi‐square test *P* value = 0.22).

### 3.3 Access to information technology and social media

Nearly all clients (321, 97.3%) had access to a mobile phone. The proportion with access to other forms of information technology and social media were as follows: Text messaging, 249 (75.5%); WhatsApp, 169 (51.2%); E‐mail, 108 (32.7%); and Facebook, 107 (32.4%). Two hundred and sixty‐nine (83.3%) clients agreed to receive health promotional messages via their mobile phones (NCD m‐Health). Their most preferred modes of messaging were phone calls (145 clients, 46.8%) and text messaging (138 clients, 44.5%). The preferred schedule for receiving such messages were monthly (181 clients, 57.1%), fortnightly (123 clients, 38.8%), or daily (13 clients, 4.1%). Overall, 125 clients (39.4%) indicated they would consider unsolicited sending of such messages to be an invasion of their privacy. This included 107 of the 125 (ie, 40.5%) clients who agreed to receive the messages.

In bivariate analysis, the sociodemographic characteristics of clients that were associated with acceptance or rejection of NCD m‐Health messages were male sex (OR = 0.44; 95% CI, 0.23‐0.86; *P* value < 0.01), aged 31 to 50 years (OR = 0.18; 95% CI, 0.08‐0.42, *P* value < 0.01), Christian religion (OR = 0.14; 95% CI, 0.02‐1.12; *P* value = 0.04), and educational level beyond JHS (OR = 3.19; 95% CI, 1.38‐7.37; *P* value < 0.01). Compared with single women, those who were married were less likely to accept m‐Health messages (OR = 0.45; 95% CI, 0.22‐0.92; *P* value = 0.03) (Table 1). Clients who were aware that obesity was a risk factor for NCD (OR = 0.40; 95% CI, 0.17‐0.93; *P* value = 0.03), those whose most recent BP check was more than 12 months ago (OR = 0.43; 95% CI, 0.20‐0.93; *P* value = 0.03), and those who had a family history of diabetes (OR = 0.33; 95% CI, 0.14‐0.79; *P* value = 0.03) were also less likely to accept NCD m‐Health (Table 2). In multivariate analysis using logistic regression, the variables that were associated with rejection of NCD m‐Health included being male (OR = 0.39; 95% CI, 0.18‐0.83), being aged 31 to 50 years (OR = 0.22; 95% CI, 0.07‐0.61), and knowing obesity to be a risk factor for NCD (OR = 0.32; 95% CI, 0.13‐0.79). In contrast, being educated beyond junior high school (OR = 4.77; 95% CI, 1.72‐13.18) was a predictor of the acceptability of NCD m‐Health. The multivariate model was significant at a *P* value of <0.01 (Tables 1 and 2).

## 4 DISCUSSION

We have used a cross‐sectional study to assess CP clients' knowledge of the risk factors for NCDs, willingness to be screened for NCDs, and willingness to be enrolled in an NCD m‐Health program. The sociodemographic characteristics of the sample surveyed is similar to that of the general population in the two districts, as established in the 2010 National Population and Housing Census.^28^ The higher proportion of males reflect the census finding of a higher male‐to‐female ratio in the urban areas of these districts. Characteristics such as age, marital status, ethnicity, and educational level generally coincide with the findings of the census.

Current estimates suggest that between 30% and 40% of the adult population in Ghana have high BP, and out of this proportion, about 30% to 40% are unaware of it.^29^, ^30^, ^31^, ^32^ In this study, we found that a high proportion of pharmacy clients (including high‐risk individuals) had neither checked their weight nor BP for over 12 months. The finding is consistent with other studies in Ghana and countries in the subregion. They help to explain why complications such as stroke have become the unfortunate events through which uncontrolled BP often gets to be diagnosed.^9^, ^33^, ^34^ The findings of this study make a case for increased access to NCD screening in this population. A policy‐guided introduction of NCD screening in CPs in Ghana will be an important addition to PHC.

In this study, we found that there is very little awareness of the fact that family history is a risk factor for hypertension and diabetes. Family history is among the strongest risk factors for the development of hypertension and diabetes.^35^, ^36^ A population‐based survey among 5389 adults in The Gambia found that a significant number of subjects with a family history of hypertension had a higher diastolic BP, body mass index, higher cholesterol, and uric acid concentrations than those without such family history.^37^ The fact that family history is a risk factor for most NCDs needs to be emphasized through public education. Given the fact that about half (50.6%) of respondents were educated beyond the Junior High School level, the finding that only 32.8% of respondents knew family history to be a risk factor for NCD is worrying. Besides access to services, this could be another important reason why 57.5% and 71.3% of respondents with a family history of hypertension had not had their weight and BP checked for over 12 months, respectively. Another risk factor that did not appear to have a bearing on the health‐seeking behavior of respondents was age. There is the need to integrate education on the risk factors for NCDs into school curricula as part of the national response to the challenge posed by NCDs. Information on how age and family history are linked to the risk of NCD should be made a standard part of NCD screening in CPs and over‐the‐counter–medicine shops.

Overall, the findings made in respect of current health‐seeking behavior contrast with the overwhelming support for the idea to introduce NCD screening into CPs. This suggest respondents had an inherent appreciation of the value of NCD screening and look forward to the opportunity to get screened. However, the fact that those who declined to participate in the survey cited time as a constraint is important. This is because when actual screening comes to be introduced, clients will be required to spend even more time at the CP. Among clients who decline to be screened due to time constraints, there may be a high proportion of those who are hard pressed for time, likelier to be under stress and, therefore, at greater risk of being hypertensive. This is likely to reduce the number of clients who will be found during screening to have hypertension or to be at risk of developing it. Serious consideration needs to be given to reducing to the barest minimum the additional time clients will spend in CPs to get screened for NCDs.

Over the many years that the health systems in LMICs have focused predominantly on the prevention and treatment of infectious diseases, insufficient attention has been given to NCDs. Primary health care programs have hardly included basic interventions like adult screening for NCDs. The fact, therefore, that a sizeable proportion of clients (including people at high risk) have not had weight or BP checked for a long time may as much be due to poor health‐seeking behavior as it may be due to an unreformed PHC system.

The detection of hypertension in Ghana remains low and haphazard.^23^, ^26^, ^31^, ^38^ This translates into a higher incidence of stroke and other complications of uncontrolled hypertension. A review of adult admissions at the Komfo Anokye Teaching Hospital, the second biggest in Ghana, found that stroke constituted 9.1% of total medical adult admissions and 13.2% of all medical adult deaths^39^ from January 2006 to December 2007. There is the need for a reform of the PHC system in the country to take into account primordial, primary, and secondary NCD prevention.

In this study, we found instances that suggest missed opportunities for NCD counseling, screening, and detection of life‐threatening situations. Extrapolated across the country and the subregion, it is conceivable that hundreds of thousands of such missed opportunities occur daily with deleterious consequences. In Ghana, the industry of CPs is driven largely by the private sector. While pharmacists and assistants are professionally enjoined to consider their work in CPs within the social context, the urge for profit could undermine a commitment to public health and PHC.^40^ Client expectation that NCD screening services should either be free or for token is unlikely to meet with the expectations of CPs. Given the overall public health good that NCD screening in CPs will serve, there is a case for services rendered to be reimbursable under the Ghana National Health Insurance Scheme. A rethinking of PHC and a firm, formal integration of community‐based NCD screening in Ghana and other countries in SSA is long overdue.^41^ Community pharmacies can play a key role in this regard.^27^

The finding in this study that over 90% of respondents have access to mobile phones is consistent with reports indicating that mobile phone voice and data penetration in Ghana stands at 127% (implies more active phones than the population) and 65%, respectively.^42^ Particularly striking for this largely rural setting is the fact that the messaging platform *WhatsApp* was available to over 50% of respondents. There is certainly in this population the opportunity to explore the use of m‐Health resources to promote NCD prevention and follow‐up on services. The diversity in preferences expressed in respect of desired frequency of messaging, the forms of messaging, and the issue of privacy‐invasion point to the fact that the use of NCD m‐Health resources suggest a need to customize services. Individual‐level informed consent and flexibility in the options available to clients needs to be considered.

In this study, we found that clients with little or no education were less likely to accept NCD m‐Health messages. This is understandable given that the ability to read and write is required for clients to independently understand NCD m‐Health text messages. Our findings are consistent with the recommendation that m‐Health interventions be designed to be easy to use by people with low educational levels.^43^ Despite great enthusiasm for NCD m‐Health innovations in SSA, there is very little routine implementation.^44^

### 4.1 Limitations

Despite some profound findings made in this study, there are important limitations that need to inform interpretation of the findings. The study was limited to six private CPs in the three districts. It is conceivable that location and customer service at selected CPs influenced attitudes towards the proposed NCD screening. Another important limitation is the small sample size. The effect of this is particularly evident in the outputs of the regression analysis where small numbers in some of the cells undermine the robustness of the established estimates. A follow‐up qualitative exploration of the findings could also have helped to contextualize some of the findings. Of interest is the role of sex. In this study, we found that female clients were less likely to accept NCD m‐Health. The role of sex in the acceptance of m‐Health needs to be explored as it has been found to greatly influence health‐seeking behavior in African rural and suburban settings.^45^, ^46^ This study was conducted among clients who were already seeking a health service. A household survey is likely to yield a less biased group of respondents.

## 5 CONCLUSIONS

The knowledge of family history as a risk factor for NCD is low in this population. There is an urgent need to promote access to screening for hypertension, especially among individuals who are at high risk because of age and first‐degree relations who have hypertension. The prospect for introducing screening for NCDs in CPs appears acceptable to most clients of CPs. Pilot implementation studies that incorporate m‐Health and involve LCS as well are needed to facilitate a learning‐by‐doing approach in the country.

## CONSENT FOR PUBLICATION

Not applicable

## AVAILABILITY OF DATA AND MATERIALS

The datasets used and/or analyzed during the current study are available from the corresponding author on reasonable request.

## FUNDING

None.

## CONFLICT OF INTEREST

The authors declare that they have no competing interests.

## AUTHOR CONTRIBUTIONS

Conceptualization: Dr. Frank Baiden, Richard Akutey

Data curation: Richard Akutey, Reina Der, Frank Baiden

Writing—review and editing: Richard Akutey, Reina Der, Frank Baiden

Critical review: Frank Baiden, Professor Frances Owusu‐Daaku

Approving final manuscript: Richard Akutey, Reina Der, Frank Baiden, Professor Frances Owusu‐Daaku

All authors approved the final version of the manuscript.
